# An Approach for Managing Manufacturing Assets through Radio Frequency Energy Harvesting

**DOI:** 10.3390/s19030438

**Published:** 2019-01-22

**Authors:** Muhammad Ashhal Tahir, Borja Ramis Ferrer, Jose Luis Martinez Lastra

**Affiliations:** Future and Automation Systems and Technologies Laboratory (FAST-Lab.), Tampere University, P.O. Box 600, FI-33014 Tampere, Finland; ashaltahir@gmail.com (M.A.T.); jose.martinezlastra@tuni.fi (J.L.M.L.)

**Keywords:** industrial automation, radio frequency energy harvesting, wireless MEMS sensors, manufacturing systems

## Abstract

The manufacturing industry requests novel solutions that will permit enterprises to stay competitive in the market. This leads to decisions being made based on different technologies that are focused on real-time accurate measurement and monitoring of manufacturing assets. In the context of traceability, radio frequency identification (RFID) tags have been traditionally used for tracking, monitoring, and collecting data of various manufacturing resources operating along the value chain. RFID tags and microelectromechanical systems (MEMS) sensors enable the monitoring of manufacturing assets by providing real-time data. Such devices are usually powered by batteries that need regular maintenance, which in turn leads to delays that affect the overall manufacturing process time. This article presents a low-cost approach to detect and measure radio frequency (RF) signals in assembly lines for optimizing the manufacturing operations in the manufacturing industry. Through the detection and measurement of RF signals, the RF energy can be harvested at certain locations on the assembly line. Then, the harvested energy can be supplied to the MEMS sensors, minimizing the regular maintenance for checking and replacing batteries. This leads to an increase in the operational efficiency and an overall reduction in operational and maintenance costs.

## 1. Introduction

The demand for highly customizable products has given rise to interconnectivity of processes, assets, and products in order to realize the trend of the so-called Industry 4.0. Principally, the interconnection of manufacturing assets is done for optimization processes, and hence, to minimize cost and improve time delays, as discussed in [1,2]. One of the most common challenges faced by manufacturing industries is a lack of availability of consistent, timely, and accurate information about manufacturing resources during the manufacturing operations. The availability of such information leads to better-informed decisions to be made. Therefore, recent work has proposed frameworks for real-time information on the tracking and integration of manufacturing resources, such as the one presented in [3,4].

Wireless sensor networks make use of wireless devices for communication, tracking, collection, and synchronization of manufacturing data. Some of the wireless devices include radio frequency identification (RFID) tags or automatic identification (AutoID) tags [5]. RFID technology is extensively used for tracking and identification of manufacturing assets, goods, and resources. RFID tags are contactless and are placed on pallets, individual parts, resources, containers, or finished goods [6]. An approach is introduced and implemented in [7] that uses RFID tags to deal with collective objects by correctly identifying and tracking them. The aforementioned approach is implemented in a context of a manufacturing system producing customized gift boxes. Furthermore, there are different applications detailing the use of RFID tags for purposes such as tracking, localization, and inventory management, as mentioned in [8,9,10]. As part of the approach, RFID tags are used to optimize the operations.

While RFID tags are widely used to collect location data, there are certain limitations associated with them. RFID tags are limited to providing location data at specific checkpoints or at periodic intervals only. In manufacturing operations, faults in production, and assembly lines lead to the products being lost track of and being damaged. There is therefore a need for continuous tracking of pallets to know the exact location of the faults in production and assembly lines. The approach in [11] introduces wireless microelectromechanical systems (MEMS) sensors. These MEMS sensors are embedded with a three-axis accelerometer and gyroscope, which provide accurate information about pallet location. However, one big challenge linked with MEMS sensors is that they are usually powered by limited energy batteries, and thus, there is always a need to replace the batteries at intermittent intervals. This regular replacement of batteries, termed in this article as regular maintenance, leads to delays in the overall manufacturing process time. Additionally, there is a risk of batteries leaking their content to the surroundings, damaging the pallets, sensors, and, in turn, the environment where human operators work. In this context, there is a clear need for proposing a solution for removing the need for batteries, as this would reduce manufacturing delays and impact on the working environment.

This article presents a novel approach that permits the minimization of the need for regular maintenance of manufacturing assets, since it will not be necessary to replace batteries of the embedded sensors, which are used for tracking pallet location. As previously presented in [12], the implemented solution allows the detection and measurement of radio frequency (RF) signals at desired locations on the factory shop floor. Existing solutions that use web services, android applications, and mobile devices for maintenance in the manufacturing domain are discussed in Section 2. In addition to some of the findings and concepts described in such solutions, the focus of this research is to provide a cost-effective approach that reduces the overall maintenance expenses without reducing efficiency. The proposed low-cost solution is based on using common devices such as smartphones for harvesting the ambient RF signals that are present indoors. Although there are numerous off-the-shelf android applications that can measure RF signal strength, they do not serve the purpose of communicating with a manufacturing system, and hence they cannot be used in the approach adopted. Besides the description of the approach and a specific proof of concept, this article provides analysis of similar solutions for RF energy harvesting. The main contribution of this article is enabling the harvesting of RF energy in large manufacturing systems. This is achieved within an RF harvester and a developed android application customized for interacting with the manufacturing system. First, the approach shows the creation of RF maps [13] of the production and assembly lines. Then, the RF maps are used for harvesting the RF signals at certain locations on assembly lines and converting them to electrical energy [14]. The harvested electrical energy from RF signals can be supplied to the wireless MEMS sensors, thus eliminating the need for the regular maintenance of embedded sensors. Furthermore, the developed android application can be used as a generic application in any web-service-based manufacturing system. Thus, the presented approach can be implemented as a quick solution for the measurement and collection of RF signal information.

The rest of this paper is structured as follows. Section 2 presents a literature review, several tools and current industrial practices used in the scope of this research. Section 3 presents the main approach components and their interrelations. Section 4 describes a case study for proving and validating the concept. Section 5 discusses different aspects of the research work, i.e.: (1) the results of the research work; (2) visualization of signal strength; and (3) RF harvesting test cases. Finally, Section 6 concludes the research work and presents future work.

## 2. Literature Review and Current Industrial Practices

### 2.1. Energy Efficiency in Factory Automation

Having a continuous source of energy is one of the critical foundations of a manufacturing industry. The manufacturing process is mainly divided into three parts: input, process, and output. The input consists of materials, information, and energy; the process consists of carrying out operations that transform a material’s shape; and, at the end, the output consists of creating a final product and waste. The portion of input energy is used for transforming the product, the majority of which is consumed for carrying out peripheral functions and the rest is transformed into waste [15]. According to [16], industrial production and manufacturing creates a substantial amount of energy demand. Most of the energy utilized originates from non-renewable sources that have price constraints. Manufacturing companies face the challenge of having a compromise between environmentally friendly operations and competitiveness. The increase in energy costs leads companies to produce products that are more expensive. Therefore, having an energy efficiency framework or energy efficient systems is very important. This not only benefits the environment, but also helps the companies to adopt sustainable production and reduce the energy usage, as mentioned in [17].

In [18], an approach is introduced that implements key performance indicators (KPIs) for measuring the energy efficiency of manufacturing operations. The KPIs are calculated for evaluating performance for on-site energy generation in four different factory levels, i.e., factory, process line, machine, and product. The KPI development process is facilitated by developing templates that can be modified to suit the specific needs as per the requirements. According to [19], the goals for monitoring and optimizing and energy efficiency are to reduce costs, energy losses, and factory or product carbon footprints, and to optimize performance. Besides this, a different approach for the real-time monitoring of energy efficiency on the factory floor by using Internet of Things (IoT) enabled software application is presented in [20]. The user, such as a floor manager or a supervisor, uses a tablet to monitor the energy performance of each machine. The energy and operation parameters are collected through sensors and production data is assessed though existing manufacturing execution systems (MES). The three components—energy manager, server, and data acquisition—work together to provide real-time monitoring of energy efficiency as well as to help identify energy gaps and abnormal energy patterns.

### 2.2. Existing Radio Frequency (RF) Harvesting Solutions and Various Approaches for Asset Maintenance

Wireless sensor networks (WSNs) refers to a collection of sensors that are distributed geographically in a physical space. Principally, WSNs are used for measuring and monitoring physical environmental conditions such as temperature, pressure, and humidity, as well as for reducing the amount of physical cables at facilities. There exists a large amount of applications of WSNs. As mentioned in [21], WSNs have found use in environmental monitoring, animal tracking and control, built environment and structural monitoring, safety, security and military applications, and health applications such as patient monitoring and drug administration.

The most common source of power for the nodes of WSNs is a battery that can either be charged or replaced, as mentioned in [22]. The limited energy source from the battery has led researchers to look for and develop alternative energy-providing mechanisms and sources. One such method of providing energy is harvesting the energy from ambient sources. Energy harvesting is described as a process of extracting energy from exterior sources, such as solar, wind, or kinetic energy, and storing it in energy storage devices such as rechargeable batteries or capacitors.

According to [23], energy for WSNs can be provided through three different methods: battery, harvesting, and transference. The energy provided based on battery is considered as the most feasible option for powering the sensor nodes that are deployed in remote regions, due to the low power requirements of the sensor nodes; however, unfortunately, in the long term, batteries always drain out. There are various ambient energy sources available for energy harvesting, such as solar, vibration, wind, thermoelectric, electromagnetic, temperature gradient, and RF. Some of the energy sources are considered periodic, as they depend on continuous provision of sources such as sunshine for solar and air for wind. Furthermore, they are impractical in WSNs due to their bulky hardware requirements, whereas energy sources based on electromagnetic, RF, and mechanical vibrations show a more constant presence. According to [24], RF energy harvesters have smaller dimensions and a more constant source of energy compared to other harvesters, and thus they are of benefit for miniature applications. In [25], an approach is introduced that uses a novel technique called magnetic resonance coupling [26] or wireless power transfer. A scenario is considered where a mobile charging vehicle travels periodically to different nodes inside a wireless network and charges the nodes of the WSN wirelessly. Furthermore, experiments revealed that the two energy storage devices do not need any physical contact for the transfer of electric power from one device to another. Moreover, as explained in [25], the distance should not be more than 2 m between the power charging and receiving nodes and with no line of sight for efficient energy transfer.

Besides this, various other technologies have been developed for RF energy harvesting. In [27], Fahira et al. integrated super capacitors in sensors of WSNs. The sensors are allowed to harvest the ambient RF energy for the operation of the WSNs. Additionally, an unmanned vehicle is installed to further facilitate the RF energy harvesting for WSNs by providing a dedicated RF energy source by using the harvesting equipment from Powercast Corp. In [28], a similar technique was used for harvesting RF energy by using Powercast products in order to activate a light fidelity (Li-Fi) sample device. In [29], Alex et al. introduced an approach that uses a radio-frequency energy harvesting system (REHS) to define specifications for a stand-alone wireless node. To harvest the RF energy, the REHS uses rectifying antenna in combination with a RF/DC converter. The harvested energy is stored in a capacitor or super capacitor. The REHS receives an input RF power of 1 mW and provides an output DC power of 0.57 mW, as represented in [29]. In [30], a new design for an RF energy-harvesting device is presented; the device has a dual-stage harvesting circuit composed of 7-stage and 10-stage design. The former design is more useful for low RF power regions (−20 dBm) and the latter is more useful for high RF power regions (+20 dBm). One of the main advantages of harvesting energy from RF signals is that RF signals are more easily available indoors than other energy sources, e.g., thermal and solar [31].

Different approaches are adopted for the maintenance of assets and resources in the manufacturing domain. For example, an approach that offers remote machine maintenance of systems through the internet by using mobile communication technologies is introduced in [32]. The approach uses XML format as the core of a remote maintenance system to encode the diagnostic data for exchanging information. Similarly, the authors of [33] discuss an Android-based smartphone system that is used for the maintenance of remote large-scale systems in the context of logistics planning. The technician uses smartphones to gather information regarding the faulty circuit boards using pictures and/or quick response (QR) codes. Afterwards, the information is checked against a database using an algorithm, assisting the technicians in repairing the faulty circuit boards. On the other hand, a dashboard that allows shop floor workers and production supervisors to visualize analytics-based information in a smart factory on a smartphone is presented in [34]. In the same scope, the authors of [35] introduce an Android mobile application to enhance productivity and efficiency in the manufacturing industry. The application is implemented within a client-server architecture and enables the users to send messages and call and view reports. Furthermore, the use of mobile Internet devices (MID) on production sites for transferring multi-source information that allows the service personnel to use the information in order to resolve issues remotely is mentioned in [36]. This permits the reduction of some maintenance costs, e.g., visits of technicians to the sites. Additionally, ‘RFTrack’ is an Android application introduced in [37]; it reads the data from a low-cost RF Explorer spectrum analyzer, geo-tags the readings, and then saves them.

## 3. Components and Interrelationships of the Approach

This section describes the components that are needed for implementing the approach and the relationship between all of them. It should be noted that not all the components of the approach interact between each other. The requirements that are needed for implementing the approach are also presented in this section.

### 3.1. Main Components for Harvesting RF Energy at Manufacturing Facilities

The approach consists of four main components: manufacturing system, smart application as the developed software, user interface, and the RF harvesting module. Aside from the RF harvesting module, the remaining three components interact with each other and are divided into different components, as described in the following sub-sections.

#### 3.1.1. Manufacturing System

The manufacturing system is mainly the system where the sensors for the traceability of manufacturing assets are deployed. The manufacturing system arranges a space for detecting and harvesting the ambient RF signals. The manufacturing system can be, e.g., any piloting line that can be used for processing the complete or partial life cycle of a product.

The manufacturing system may consist of a combination of workstations or work cells connected with conveyors that can be reconfigured in different configurations. In a particular arrangement, a material can be loaded on one or more workstations. Every workstation offers a unique operation that can be carried out on the material, and all workstations work together to create a final product before it is unloaded at one of the workstations for delivery to the customer. The material passes from one workstation to another with the help of pallets moving on conveyors. The specific arrangement of workstations and the movement of pallets through them determines a unique path for pallets. The presence of RF signals on this unique path can be detected and their signal strength measured. This will allow the creation of a signal strength map of the path that can help identify potential locations for harvesting the ambient RF signals and converting them to energy for powering the wireless sensors.

In large manufacturing systems, multiple tests can be carried out based on different RF energy sources, and then the results can be compared together. This will help to identify the best harvesting locations on the path and furthermore help identify the best performing RF energy sources.

#### 3.1.2. Smart Application

**Figure 1:**
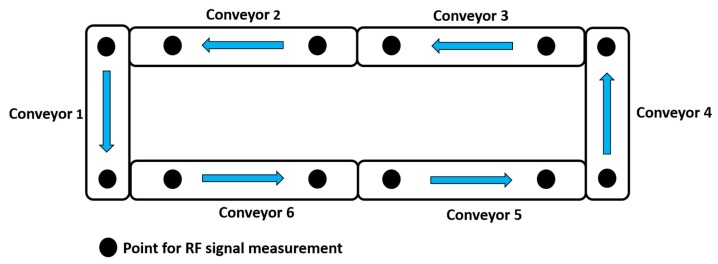
A suggested RF map.

Smartphones contain sensors that can detect RF signals based on different frequencies. In order to utilize the sensors of a smartphone, a software application is required. To create an RF map of the path based on pallet movement, as suggested in Figure 1, a smartphone is placed on top of the pallet that consists of embedded wireless sensors. As the pallet traverses the path, the Android application detects the presence of ambient RF signals in the environment and measures their strength. Furthermore, the Android application can send data to an application programming interface (API) that can transfer the RF signal parameters to a user sitting in a remote location.

The Android application is developed keeping in view the requirements of the manufacturing system. The requirement necessitates the manufacturing system to have conveyors that can be connected with each other in different configurations, pallets capable of moving products on the conveyors, wireless sensors used for keeping track of pallets, and web-service-enabled controllers to control different segments of the manufacturing system. Additionally, the software application needs to be interfaced with the information system of the manufacturing system in order to know the exact location of the pallet on the conveyor path.

#### 3.1.3. User Interface

The user interface is divided into two different parts: user interface of the smart device and web-service endpoint. The user interface of the smart device is where the RF signal parameters can be viewed in real-time when the application is not being utilized. On the other hand, the second user interface is accessible through a web service at a remote location. With the help of an API, the Android application stores the RF signal information in a database. The web service extracts the RF signal parameters from a database and provides them on the user interface for the user to visualize.

#### 3.1.4. RF Harvester Module

**Figure 2:**
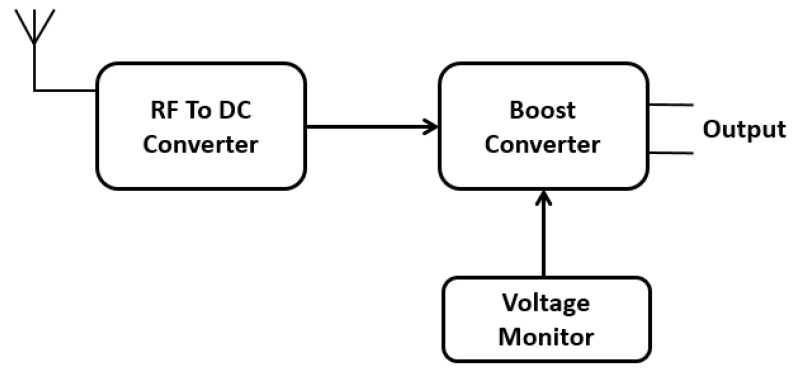
RF harvester block diagram.

An off-the-shelf RF harvester module is utilized to harvest the ambient RF signals present in the path defined by the pallet. To validate the results, the RF harvester is used to harvest the RF signals on various locations of the identified path in conveyor and the harvested energy is stored in a capacitor. Figure 2 shows the block diagram of the RF harvester, showing its main functionality. The input to the RF harvester is received as RF signals from the antenna. Then, there is a conversion from RF to DC signals. As the DC signals are weak, they are amplified within the boost converter in the next stage to make them useful. The voltage monitor constantly monitors the DC power and allows the user to know when a certain level of voltage is present. The boost converter is used to power up the capacitor and the stored charge in the capacitor can be used at the user’s discretion. The RF harvester is further used for various testing approaches, which are discussed in the results section.

### 3.2. Interrelationships between the Components of the Approach

**Figure 3:**
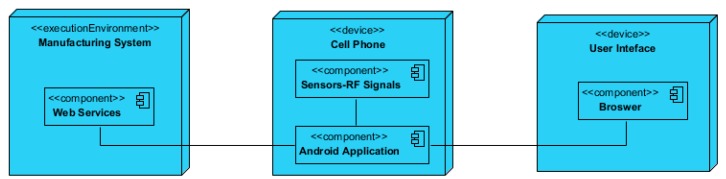
Interrelation of components.

From a communication perspective, the manufacturing system, software application, and user interface are interconnected with each other through different protocols, as shown in Figure 3. The manufacturing system should include a set of industrial controllers with enabled web-service technology in order to allow its interaction with the smart application on the smartphone. Moreover, the smartphone contains embedded sensors that are used for detecting and measuring the ambient RF signal strength. The application is connected with the user interface through the web browser. In this manner, the Android application sends the RF signal parameters, which can be viewed by the user in a remote location.

The fourth component, i.e., the RF harvester module, is not presented in Figure 3, as it is an independent component that does not communicate with the rest of the components. The RF harvester module is uniquely used for testing the harvested signals at various points in the pallet path.

## 4. Proving the Concept: The FASTory Assembly Line as a Testbed for RF Energy Harvesting

This section describes in detail the implementation of the approach from both hardware and software levels. The section discusses the hardware components used and their characteristics and gives a detailed description of a developed Android application and representational state transfer (REST) API. Finally, it describes the interaction between the modules, including the selected tools and protocols for communication.

### 4.1. The FASTory Assembly Line

**Figure 4:**
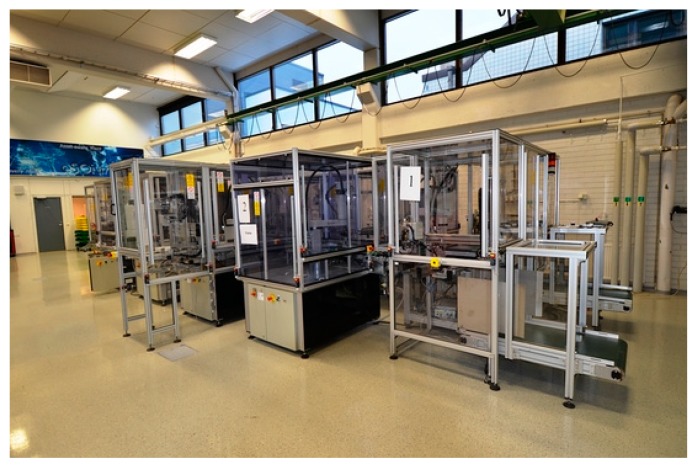
The FASTory assembly line.

The FASTory assembly line was selected as the testbed for the implementation of the approach. This system is located at the Factory Automation Systems and Technologies Laboratory (FAST-Lab), which belongs to the Tampere University of Technology in Tampere, Finland. The FASTory assembly line can be seen in Figure 4.

The FASTory assembly line consists of 12 workstations (WS), which are labeled as WS1, WS2,…, WS12. WS 1 and 7 are used for loading and unloading pallets and papers. Then, WS2–WS6 and WS8–W12 are equipped with a four-axis selective compliance assembly robot arm (SCARA) SONY SRX-611 robot. All the workstations are connected with each other through conveyors in a rectangular format with a bypass channel for the purpose of line balancing. Additionally, the conveyors are equipped with NFC/RFID readers, safety systems, and feeders.

Each component of the conveyor and all robots are supplied with a remote terminal unit (RTU) known as the S1000. The Inico S1000 is a web-service-enabled technology RTU capable of processing field data in real time. It offers web-based monitoring and can be integrated with enterprise IT or supervisory control and data acquisition (SCADA) systems. The Inico S1000 is designed and manufactured by Inico Technologies (http://www.inicotech.com/) and uses web services and other built-in apps that can be programmed and configured with various web browsers.

The S1000 controllers are deployed in the FASTory assembly line and are responsible for managing different segments of the assembly line such as robotic arms and conveyors. Additionally, various expansion modules can be used with S1000 that can permit special functionality such as wireless networking, communication, and energy monitoring, as described in [38].

**Figure 5:**
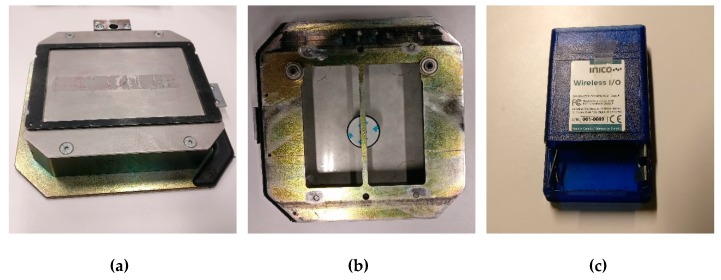
(**a**) The top flat surface of a pallet. (**b**) The hollow bottom of the pallet, containing a Radio Frequency Identification (RFID) sensor. (**c**) Wireless embedded sensors which are placed in the hollow bottoms of the pallets together with the RFID sensor.

The pallets are transported from one workstation to another in order to pass by the working positions of the required workstations. In terms of structure, the pallets are divided into two parts that consists of lower part made of a steel frame that acts as an interface between the conveyor system and the belt. The frame has small metallic wheels that allow the pallets to travel on the conveyor belts as seen in Figure 5b. A hollow box is mounted on top of the steel frame, and contains embedded wireless MEMS sensors below, and, on the top surface of the box, a detachable plate used for placing the materials or products. To allow the tracking and scheduling of pallets, they are equipped with circular RFID tags. Each pallet has a unique passive RFID tag that is used to assign a distinct identification to the pallet through which it can be identified. The conveyors contain near-field communication (NFC) readers that read the passive RFID tags inside the pallets and their unique IDs. Such applications of RFID and NFC technology are explained in further detail in [39].

Furthermore, two other components are used with the FASTory assembly line, namely the MEMS sensors and RF harvester module. The embedded MEMS wireless sensors consist of a gyroscope and an accelerometer that are located inside the pallets. These embedded wireless sensors are designed and manufactured by Inico Technologies Ltd. (Calgary, AB, Canada). The gyroscope and accelerometer are used for tracking the location of the pallets. The location parameters from the embedded sensors are accessed via web services. The wireless sensors are powered by two AA batteries, run in two different modes—run or configuration mode—and operate at a frequency of 2.4 GHz for communication, as discussed in [11]. The RF harvester module used in this research work is P21XXCSR-EVB, evaluation board from Powercast [40]. It takes an input RF signal in the range of −15 dBm to +15 dBm, and depending on the input signal strength, the output voltage can be anywhere between 0.7 and 1.2 volts. The RF harvester module is described in further detail in Section 5.

### 4.2. Component Implementation

For implementing the approach, REST has been selected in order to develop web services offering interoperability for the exchange of information and data between systems. The REST web services offer a predefined set of operations for accessing the textual representation of the web services. The FASTory assembly line is connected with RTUs that serve both REST [41,42] and Simple Object Access Protocol Devices Profile for Web Services (SOAP DPWS) [43] services. Although S1000 offer both REST and DPWS services, only the REST interface is used in this research.

On the other hand, the Android Studio software was chosen for developing the Android application. Android Studio was selected due its open-source nature, access to thousands of tutorials, and development support. It is easy to use, developed applications can be easily customized, and it is compatible with a range of smartphones and other devices. Furthermore, the developed Android application can be smoothly integrated with the APIs, databases, and REST web services.

As described in [44], the implemented approach can be viewed in the form of a design pattern that mimics the model–view–controller (MVC) pattern. This allows the proposed solution to be divided into three interconnected modules, allowing for the efficient reuse of code and parallel development of these modules.

- Model: the model is defined as the central component of the interface. It is used for handling the algorithm, data structures, and logic. In the proposed solution, the model can be taken as an android application. The android application obtains the data from sensors and processes it for various purposes.
- View: the view refers to the visualization and representation of the information that can be viewed and manipulated by the user. The view in the proposed solution can be seen as the graphical user interface (GUI) through which the user can monitor and view the information.
- Controller: The controller bridges the model and view, thus allowing the flow of information among them. The controller in this case is the REST API that establishes communication between numerous components operating on different protocols.

**Figure 6:**
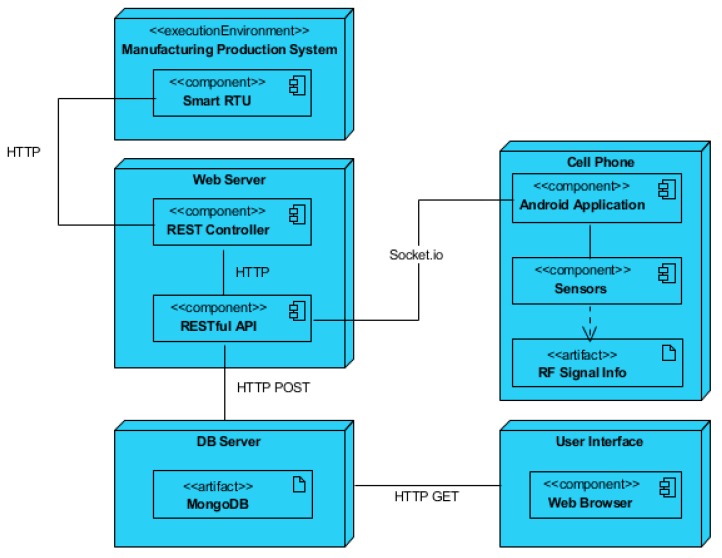
Architectural view of implementation of the approach.

The proposed solution is implemented based on the architectural view shown in Figure 6. The proposed software solution developed mirrors the MVC approach, as discussed above, and shows interconnection between the Android application, FASTory assembly line via RTUs and user interface.

As seen in the Figure 6, the smart RTU is connected with the robotic arms in the workstations and conveyors. The RTU can be accessed through web services based on HTTP protocols. A server is created that contains the REST controller and the REST API. The REST controller is used for invoking services from the FASTory assembly line, which enables physical actions on the assembly line such as moving the pallet from one station to another while the smartphone is placed on top of the pallet or activating robotic arms to perform a certain task. The REST API receives signal parameters from the Android application based on socket programming. The REST API then stores the received signal parameters on database. The user interface extracts the relevant information from the database and displays it in the web browser for the user to view. The Android application is installed on the smartphone, which is placed on top of the pallet as it moves through the defined path on conveyors. The embedded RF sensors inside the smartphone measure the signal strength at various checkpoints on the conveyor.

### 4.3. RF Signal Parameters

The RF signal sensors embedded inside the smartphone are capable of measuring ambient RF signals from different RF sources. These RF sources include Wi-Fi access points or routers and Global System for Mobile communication (GSM) signals emanating from the cellphone base stations or towers. The RF signals from cellphone base stations have a significant presence in the environment and under certain conditions are a good source of RF signals for energy harvesting. The Android application offers two different modes: in the first mode, it can detect the RF signals from Wi-Fi or access points that have a standard signal frequency of 2.4 GHz and 5 GHz; in the second mode, it can detect the RF signal parameters from the GSM signal strength accessed via the SIM card present inside the smartphone. The GSM frequency band has a range of 800–2000 MHz. The Android application further allows the user to view RF signal parameters in real time.

The experiments conducted during the research work were carried out in a laboratory in the Tampere University of Technology. Thus, all the cellular-based frequencies used for this research are among the cellular frequencies that are currently deployed in Finland. There are different frequency bands deployed in Finland; the major ones are the 3G UMTS band B1 (2100 MHz) and B8 (900 MHz). The Long-Term Evolution (LTE) bands that are deployed consist of B3 (1800 MHz), B7 (2600 MHz), B20 (800 MHz), B1 (2100 MHz), B28 (700 MHz), B38 (2600 MHz), and B31 (450 MHz). Different cellphone carriers make use of different frequency bands and use different combinations [45]. When measuring RF signals, various signal parameters are measured besides the signal strength. Some of these signal parameters include speed, strength, unique ID, identifier, country information, network operator, data state, frequency, etc. The parameters vary depending on whether the RF source is an access point or GSM signals from a particular network operator. The list of RF signal parameters measured from an access point and a smartphone can be seen in Table 1.

### 4.4. Interfacing with Manufacturing System

#### 4.4.1. Flow of Information

The smartphone needs to be interfaced with the manufacturing system to create the RF map of the assembly line. This is done via the Android application on the smartphone that connects with the RTU of the assembly line. The web server contains the REST controller and the REST API. The REST controller is used for subscribing events of the assembly line. The subscribing of events is helpful as whenever a pallet is moved from one zone to another on the conveyor a notification is received. This helps keep track of the location of the pallet on the assembly line. The REST API is used for carrying out operations on the data received and is discussed in detail later.

**Figure 7:**
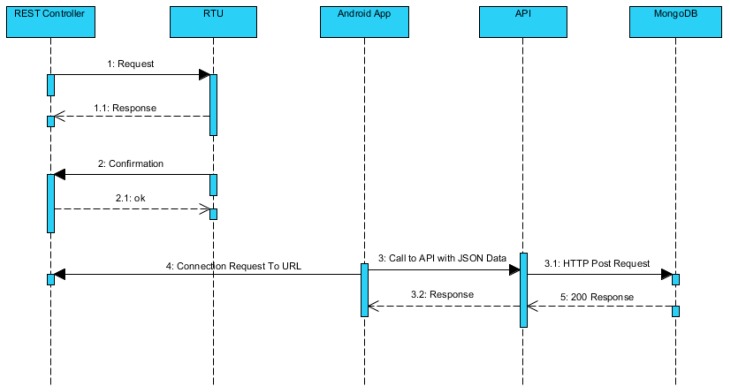
Sequence diagram presenting flow of communication.

Figure 7 represents a holistic view of the flow of information between the entities involved. Initially, the REST controller makes a request to the RTU that is confirmed by the RTU by sending a response back to the REST controller. In the second step, the smartphone needs to be connected with the manufacturing information system. This is carried out by prompting the user through the Android application interface to enter the relevant address and port. After entering the relevant information, the user presses the ‘OK’ button; this enables the connection to be established between the Android application and the manufacturing information system. Finally, the REST API is called that receives the RF signal parameters from the Android application and saves them in the database.

#### 4.4.2. Invoking and Subscribing Events

The request made by the REST controller to the RTU of the conveyor can be either a GET or POST request. The HTTP POST request is made whenever a user wants to perform a certain operation, such as moving the pallet from one zone to another. Once the operation has been carried out, it is important to know if the pallet has reached to its destination zone or not. In order to verify the presence of the pallet at the destination zone, REST controller subscribes to the event notification. This is carried out by using the POST request with a particular event ID. Similarly, the REST controller subscribes to events of all the zones present on the conveyors in the assembly line. Thus, whenever a pallet reaches a particular zone, a notification is issued that will verify the presence of the pallet in that particular zone.

#### 4.4.3. REST API

Many web-based applications need and use HTTP requests to perform operations. A simple REST API [46] is created using Node.js and Mongo DB. Node.js (https://nodejs.org/en/) is selected for creating the API since it is simple to use, works well with networking applications, and is lightweight and faster in execution. The REST API uses four operations that are termed CRUD and are commonly used in REST based architecture. The REST API is tested with Postman API (https://www.getpostman.com/) development environment to check its functionality before integrating it with the server for communicating with the Android application.

### 4.5. User Interface

The Android application has a user interface designed to allow effortless visualization of the RF signal strength and other parameters. Two different interfaces are designed that allow the user to visualize RF signal strength from access points and cellphone SIM card. The strength of the RF signals is consistently changing, and the changing signals are updated in real time in the visualizations.

**Figure 8:**
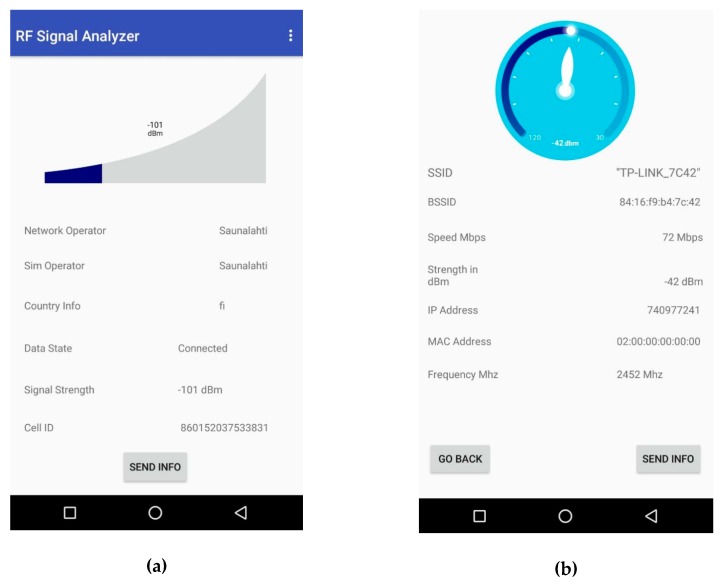
(**a**) The ‘RF Signal Analyzer’ application displays Global System for Mobile Communication (GSM) signal parameters of smartphone. (**b**) The visualization consisting of RF signal parameters from an access point.

Figure 8a displays the user interface for visualizing the RF signal strength from the smartphone. In the interface, a graph can be seen that displays the current signal strength in dBm, and additional RF signal parameters can be seen below. These parameters include the name of the operator, data state, country information, and the unique cell ID. The ‘SEND INFO’ button at the bottom of the screen enables the transfer of the data represented in the interface to the web server. After pressing the ‘SEND INFO’ button, the pallet starts moving. Then, the Android application sends the data automatically whenever the pallet reaches the checkpoint of a particular workstation.

Figure 8b represents the RF signal strength from an access point. The signal strength is displayed in the form of a speedometer with additional signal parameters displayed below. The ‘GO BACK’ button allows the user to go back to the home screen of the application and the ‘SEND INFO’ button works in a similar way as it did in the interface shown in Figure 8a. The user has to press the ‘SEND INFO’ button initially once, and subsequently data is sent automatically whenever a pallet reaches a particular zone on conveyor. The signal parameters are sent to the web server, which saves them in the database to be retrieved later for different uses.

## 5. Results and Discussion

This section analyses the outcomes from the previous section and presents additional tests based on such information. The discoveries made through the implementation and tests are discussed in this section. The section includes the RF signal survey of the premises of the FAST-Lab, a comparison of different RF harvester modules, and testing of RF harvester and power calculations.

### 5.1. RF Signal Survey

**Figure 9:**
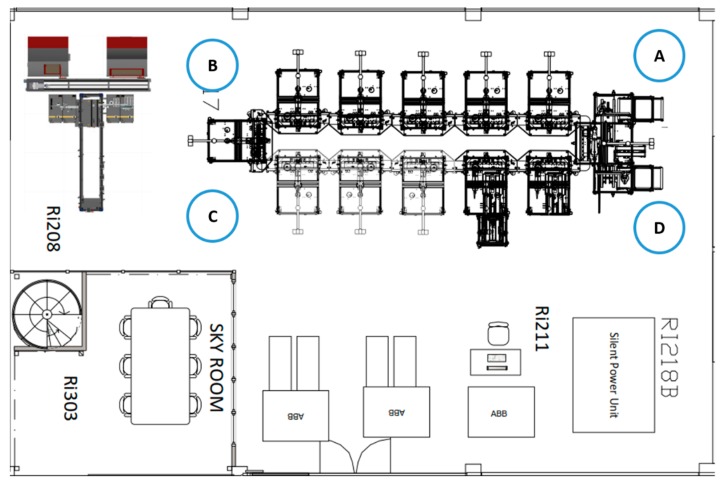
Layout of the Future Automation Systems and Technologies Laboratory (FAST-Lab). A, B, C and D are the four selected positions for measuring the RF signal strength.

The Android application is used for measuring the ambient RF signal strength in the FAST-Lab facilities, which are shown in Figure 9. In the layout, the FASTory assembly line is placed at the top right corner.

Four different positions are selected around the FASTory assembly line—labeled as A, B, C, and D—for measuring the maximum and the minimum signal strength of the RF signals. This information helps to determine the range of the RF signal present in the ambient environment. The range of the RF signals helps in adjusting the operating frequency of the antenna needed for the RF harvester. Table 2 displays the minimum and the maximum RF signal strength from three different cell operators offering services in Finland, i.e., Elisa, DNA, and Telia. The signal strength from different cell operators lies in a similar range; this is because the test points are only separated by a few feet and are located at the same laboratory. Thus, the signal strength and signal reception are alike in all the four reception test points. The minimum signal strength is measured by keeping the smartphone to the 3G mode and the maximum signal strength measured by putting the smartphone to 4G mode. Although it is not the focus of this research, it is important to highlight that the main differences between 3G and 4G are their technologies; 3G uses technologies such as EV-DO, WCDMA, and HSPA among many other. Whereas 4G uses technologies such as WiMax, LTE, and UMB. Furthermore, 4G has a speed that ranges from 100 Mbps to 1 Gbps, whereas 3G has a download speed of 14 Mbps and uplink speed of 5.8 Mbps, as mentioned in [47]. When the smartphone runs in GPRS mode, the signal strength is low and no RF signal is harvested. Therefore, the RF signals are harvested only when the smartphone is in either 3G or 4G mode with a higher signal strength.

### 5.2. Analysis of RF Harvester Solutions

There are many RF solutions and approaches available, however not all the solutions can be implemented for use in the case study of this research. Thus, it is important to evaluate all the options available in order to select the best available solution to use. However, the majority of the RF solutions available are in the research phase and are the direct result of the research work carried out. This research work considers. Powercast (https://www.powercastco.com/) and e-peas (https://e-peas.com/) as the main companies producing commercial RF products and solutions to be compared. The features of these RF products are presented in Table 3.

The requirement for the case study entails the energy to be harvested from ambient RF signals. The voltage requirement for the wireless embedded sensors is 3 volts. The energy should be harvested centered on the frequency range of RF energy sources, available in the ambience of the FAST-Lab. Thus, based on the analysis of the RF solutions present, the Powercast P21XXCSR-EVB (Powercast Corporation, Pittsburgh, USA) was selected for harvesting the RF signals. This is due to the fact that it can harvest energy from six different sources, provides a voltage up to 5.5 volts, and has a higher rating for a current that will be helpful in providing power to the wireless sensors.

**Figure 10:**
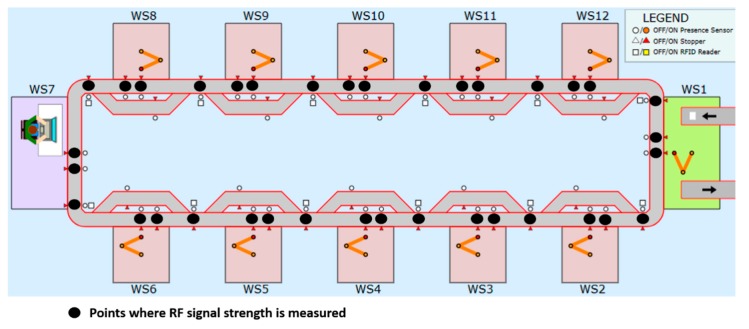
RF signal map.

The Powercast harvester is placed on the pallet together with the smartphone. The pallet is then allowed to move through a defined path, and the Android applications takes RF measurements at different zones on the conveyors of the FASTory assembly line, as seen in Figure 10. As the pallet moves, the RF harvester works simultaneously to harvest the RF signals emanating from the smartphone. Regarding various RF sources present in the FAST-Lab, the RF harvester was able to harvest RF signals from smartphones only. The Wi-Fi routers and access points are another good source of RF energy. However, the energy could not be harvested from access points or Wi-Fi routers due to them being fixed on the walls and there being a large distance between them and the FASTory assembly line; this rendered them not useful for the current scenario.

### 5.3. Test Cases

The RF harvester and smartphone were placed in different orientations, and the energy harvested was then measured for those orientations. This helped in determining the optimal position for placing the RF harvester on pallets while they are moving on the conveyors. Four different orientations were selected for testing the RF harvester and discovering the best orientation among them.

**Figure 11:**
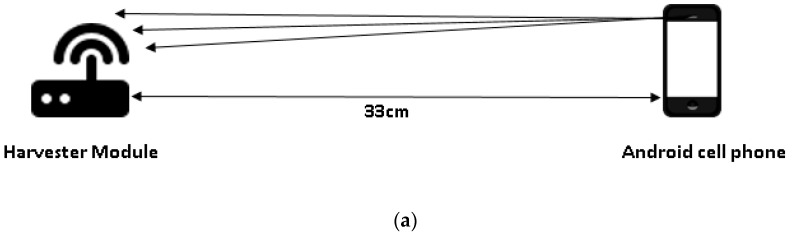

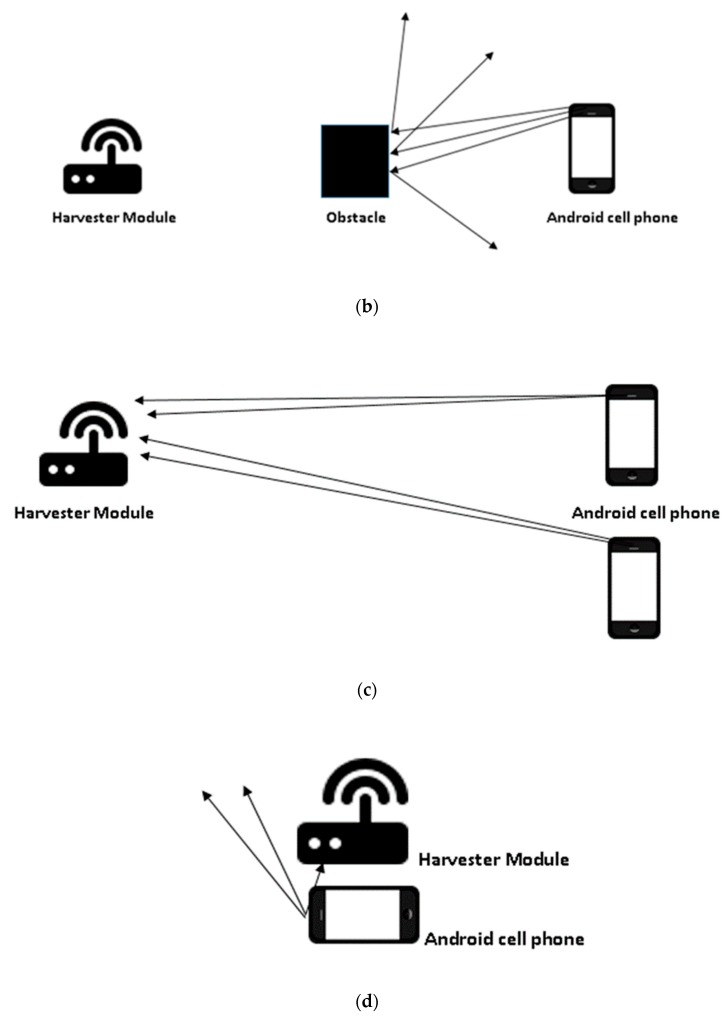
(**a**) Test case 1. (**b**) Test case 2. (**c**) Test case 3. (**d**) Test case 4.

In the first test case, the RF harvester and the smartphone were placed in each other’s line of sight, as seen in Figure 11a. An omnidirectional antenna was placed on the RF harvester, and the smartphone was rotated around the RF harvester in a horizontal plane so that the RF signals from the smartphone fell within the radiation pattern of the RF harvester antenna. The RF harvester contains a test LED that lights up when the capacitor is charged to a certain limit. The test LED is connected with the capacitor on the RF harvester module and the blinking rate of the LED is directly proportional to the charging of capacitor. The RF harvester contains a RF to DC converter that converts the RF signals to DC voltage. In the next step, the boost converter boosts the DC voltage and stores the charge inside the capacitor. It is observed that if the distance between the smartphone and RF harvester is reduced, the capacitor charges faster, as is evident from the faster blinking rate of the LED. This observation verifies the inverse square law of electromagnetic radiation [48]. From experimentation, it was determined that the maximum distance between the RF harvester and smartphone at which the LED keeps blinking is 33 cm. At any distance further than 33 cm, the LED turns off, as the intensity of the RF signals are not strong enough to charge the capacitor that in turn turns on the LED.

The frequency at which the smartphone is operating is approximately 900 MHz, as measured by using an independent mobile application called LTE Discovery (https://play.google.com/store/apps/details?id=net.simplyadvanced.ltediscovery&hl=en). There is no method to control the transmit power and the frequency of the smartphone. In order to determine the RF parameters for the smartphone antenna, the LTE Discovery application is used to measure the frequency at which the smartphone antenna operates. The manufacturing company does not disclose information regarding the antenna gain of the smartphone, and thus a reasonable estimation is made for the antenna gain. The minimum and maximum values used for the antenna gain are given in Table 4. The RF harvester P21XXCSR-EVB provides 2 volts in the output when the RF input is between the range of −15 to +15 dBm. However, the RF input provided by the smartphone is well below −15 dBm, and thus is not enough to generate a voltage of 2 volts. Additional tests were conducted by placing the smartphone at different multiples of the minimum distance of 33 cm, and no changes were observed in the harvested voltage.

In the second test case, as seen in Figure 11b, an obstacle is placed between the RF harvester and the smartphone. It is observed that the obstacle considerably blocks and scatters the RF signals from the smartphone in the environment. Hence, very little or no RF signal reaches the RF harvester. With little RF signal reaching from the smartphone, the capacitor charges up very little, and that charge is not sufficient to light up the LED. Additionally, when the obstacle is removed, the path for the RF signals is cleared, the capacitor starts charging, and eventually the LED starts blinking. 

In the third test case, as seen in Figure 11c, multiple RF signal sources are used, i.e., two smartphones are placed next to each other. The smartphones are in the line of sight of the RF harvester and are placed at the same distance from it. During the observation, no significant changes are perceived; the capacitor charges in the same amount of time as it does with one RF source; thus, the LED blinks at the same rate as well. It is therefore concluded that using multiple RF sources has little or no effect on the RF harvester.

In the fourth test case, the RF harvester is placed on top of the smartphone. As seen in Figure 11d, this placement considerably blocks the path of RF signals emitted from the smartphone. The amount of RF signal reaching the RF harvester module is much less, and the charge inside the capacitor is not enough to light up the LED.

In conclusion, the distance between RF harvester and smartphone cannot be more than 33 cm. If the distance is more than 33 cm, the RF harvester does not harvest any RF signals. Moreover, a significant loss of energy to the surroundings is observed, as the maximum harvested voltage is only 0.78 volts, which is far less than the required voltage of 3 volts for powering the wireless embedded sensors. The tested RF source is not reliable, as many variations in the intensity of the RF signals are observed. In order to harvest the required voltage of 3 volts, a dedicated and strong RF signal source is needed with a directional antenna. Thus, the first test case ranks the best among all of the test cases. Hence, all the other measurements on the conveyors are made using the orientation mentioned in the first test case.

### 5.4. Power Calculations and Energy Harvested

In order to determine the equivalent amount of energy harvested in terms of the power of the RF signals, certain calculations are performed. The Friis transmission equation (1), discussed in [49], is utilized for calculating the transfer of power from smartphone to the RF harvester. For this purpose, antenna parameters for both RF harvester and smartphone need to be known. These parameters can be seen in Table 4. It is important to note here that, due to the unavailability of exact information on the smartphone antenna, an estimation is made for the antenna gain and power transmitted. These estimations are mentioned in terms of maximum and minimum and are used in calculations.

(1) $$P_{r}=\frac{P_{t}G_{t}G_{r}\lambda^{2}}{\left(4\pi R^{2}\right)}$$

where:

$P_{r}$ is the received power
$P_{t}$ is the transmitted power
$G_{t}$ is the gain of the transmitting antenna
$G_{r}$ is the gain of the receiving antenna
λ is the wavelength of the RF signals
R is the distance among the antennas

Using the minimum and maximum values of the antenna gain defined in Table 4, the power received at the RF harvester is calculated as follows:

- Where the frequency is 925 MHz, the distance between antennas is 0.33 m, the antenna gain is 0 dB, the power transmitted is 30 dBm, and the power received at the RF harvester module is 7.71 dBm.
- Where the frequency is 960 MHz, the distance between antennas is 0.33 m, the antenna gain is 3 dB, the power transmitted is 33 dBm, and the power received at the RF harvester module is 13.40 dBm.

**Figure 12:**
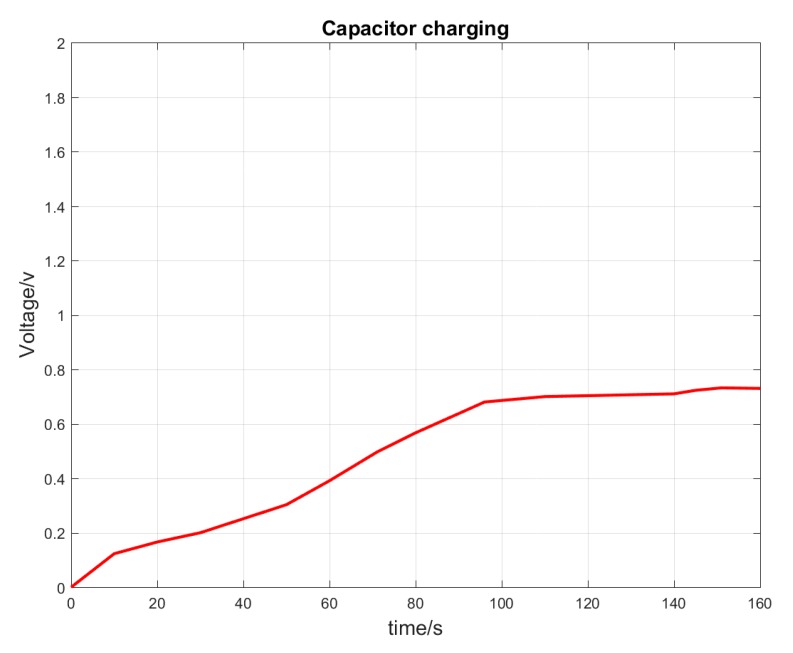
Voltage vs time for capacitor charging.

Based on the calculations, the power received at the RF harvester lies between 7.71 dBm and 13.40 dBm. The range mentioned in the theory is roughly estimated to be between 0.5 and 2.0 watts, which is comparable to the average power of RF signal waves of the GSM network. Additionally, Powercast calculator [50] is also utilized to further support in estimating the calculations. However, in reality the power received by the RF harvester is much less than the power calculated above. Based on the voltage measured from the 2200 µF electrolytic capacitor on the RF harvester board, the harvested voltage only reaches a maximum of 0.78 volts, as seen in the capacitor charging graph in Figure 12. This is mainly due to the losses to the environment based on different factors and low RF input signal.

## 6. Conclusions and Future Work

The Android application developed has been customized and designed for the FASTory assembly line. However, the application itself can be used independently for measuring RF signals from various access points and cell operators present in the environment. In fact, while this research work proposed an Android development in the proof of concept due to the selected smartphone, the approach can also be implementable with other operating systems. The Android application can only measure RF signals from GSM bands as mentioned previously in [45] as well as 2.4 GHz and 5 GHz, which correspond to the frequency of the Wi-Fi routers. Thus, any other RF signal source besides the ones mentioned above is not detected and measured by the Android application.

Regarding the functionality of the RF harvester, it can be concluded, as seen in test case 1, that it is required to have a line of sight for harvesting the RF signals into energy. If there is no line of sight, the harvested energy will not be enough to light up the LED.

The Android application was developed using Android Studio, which is an open source platform. However, the Android application can be extended for use in any other manufacturing platforms or physical environments. The Android application can be equipped with additional features; for example, it can be integrated with maps to identify the location of nearby phone towers. This will be useful when multiple phone towers are present and the user needs to verify the source of the RF signals being received.

This research has successfully identified and measured RF signal strength and harvested energy from RF signals, as seen in the implementation and results section. This is critical for reducing the maintenance of wireless MEMS sensors at periodic intervals. Then, as the time required for battery replacement is removed, the overall manufacturing process time will not be affected by such delays which interrupt the movement of pallets on the assembly line.

Future research work will focus on measuring and testing with various high-power RF sources. The authors expect to harvest energy at up to 3 volts or more from this.

## Figures and Tables

**Table 1:** Radio Frequency (RF) signal parameters.

RF Signal Source Signal Parameters	Access Point Value	RF Signal Source Signal Parameters	Smartphone Value
SSID	ashh	Network Operator	Saunalahti
BSSID	e6:db:30:01:7f:31	SIM Operator	Saunalahti
Speed Mbps	72 Mbps	Country Info	fi
Strength/dBm	−32 dBm	Data State	Connected
IP Address	1250535616	Signal Strength/dBm	−86
MAC Address	02:00:00:00:00:00	Cell ID	860152037533831
Frequency MHz	2437 MHz	SIM Operator	-

**Table 2:** RF signal strength survey.

	Elisa		DNA		Telia	
Test Point	Min Signal Strength (dBm)/3G	Max Signal Strength (dBm)/4G	Min Signal Strength (dBm)/3G	Max Signal Strength (dBm)/4G	Min Signal Strength (dBm)/3G	Max Signal Strength (dBm)/4G
**A**	−107.34	−104.36	−103	−105	−108	−109
**B**	−103.35	−104.36	−105	−106	−106	−108
**C**	−104.36	−107.33	−100	−104	−105	−106
**D**	−102.38	−102.37	−101	−107	−109	−111

**Table 3:** Comparison of Powercast and e-peas RF harvesters.

	Powercast		e-peas	
**Model Number**	**P21XXCSR-EVB**	P2110-EVB	AEM40940	AEM30940
Function	RF to DC converter	RF to DC converter	RF to AC converter	RF to DC converter
RF Input Min (dBm)	−15	−15	−19.5	−18.5
RF Input Max (dBm)	+15	+15	+10	+10
Frequency Bands Supported (MHz)	824–849	850–915	868, 915, 2450	868, 915, 2450
	865–894			
	880–928			
	1710–1785			
	1850–1910			
	2400–2500			
Output Voltage Min (V)	2.0	N/A	1.2, 1.8	1.2, 1.8
Output Voltage Max (V)	5.5	5.25	2.5, 3.3	4.1
Storage Elements	Three capacitors	Two capacitors	Five capacitors, two inductors	Five capacitors, two inductors
Max Current (mA)	50	N/A	80	80

**Table 4:** RF harvester and smartphone antenna and power parameters

	Cell Phone	RF Harvester
Parameters	Values	Values
Model Number	OnePlus 3 Graphite (EU)	P21XXCSR-EVB
Min Antenna Gain (dB)	0	0.15
Max Antenna Gain (dB)	3	-
Frequency (MHz)	E-GSM-900 (925–960)	890–960
Min Power Transmitted (dBm)	30	-
Max Power Transmitted (dBm)	33	-
